# A system solution for a 100 kA class high temperature superconducting line for HL-LHC and for wider energy applications

**DOI:** 10.1038/s41598-025-08543-9

**Published:** 2025-07-01

**Authors:** Amalia Ballarino, Wendell Bailey, Christian Barth, Paul Cruikshank, Vanessa Gahier, Yann Leclercq, Florian Pasdeloup, Gerard Willering, Yifeng Yang

**Affiliations:** 1 CERN, European Organization for Nuclear Research, Geneva, 1211 Switzerland; 2 SOTON School of Engineering, Faculty of Engineering and Physical Sciences, University of Southampton, Southampton, SO17 1BJ UK

**Keywords:** Engineering, Physics

## Abstract

The powering of the High Luminosity magnets of the Large Hadron Collider relies on Cold Powering Systems incorporating direct current superconducting lines, called Superconducting Links, based on magnesium diboride cables. A Cold Powering System interconnects the magnets in the accelerator existing tunnel to the power converters in newly excavated galleries that are about 8 m higher than the accelerator tunnel and up to about 100 m distant from the magnets. It feeds circuits rated at different currents and is designed to transfer a total current of up to |117| kA with magnesium diboride and Rare-Earth-Barium-Copper-Oxide technologies. After about ten years of development, the first Cold Powering System was successfully constructed and tested at CERN. The Superconducting Link was measured in a geometrical configuration that included a vertical path simulating the final routing in the accelerator underground. The test campaign validated the mechanical, cryogenic and electrical performance of the system both in steady state conditions and under various transient scenarios. This paper reports on the results of the tests and details the performance of the first ever built magnesium diboride and Rare-Earth-Barium-Copper-Oxide 100 kA class superconducting system.

## Introduction

The High Luminosity Large Hadron Collider (HL-LHC)^1^ is an upgrade of the LHC machine which aims at achieving instantaneous luminosities a factor of 5 to 7.5 larger than the LHC nominal value. This will allow attaining an integrated luminosity around ten times higher than the expected luminosity reach of the LHC after about 10 years of operation, and it will therefore bring new opportunities for physics discoveries. Planned to be operational as from 2030, the HL-LHC will rely on key innovative superconducting technologies. The final focusing magnets in the interaction regions close to the ATLAS and CMS experiments will consist of niobium-tin (Nb_3_Sn) quadrupoles reaching peak fields at operation of about 12 T. Superconducting radio-frequency “crab” cavities will tilt the particle beams to enlarge the overlapping area of the incoming particle bunches. Finally, the electrical transfer from the power converters to the magnets will be done via innovative Cold Powering Systems that feature direct current (DC) superconducting lines, known as Superconducting Links, based on magnesium diboride (MgB_2_) technology^2^.

**Fig. 1:**
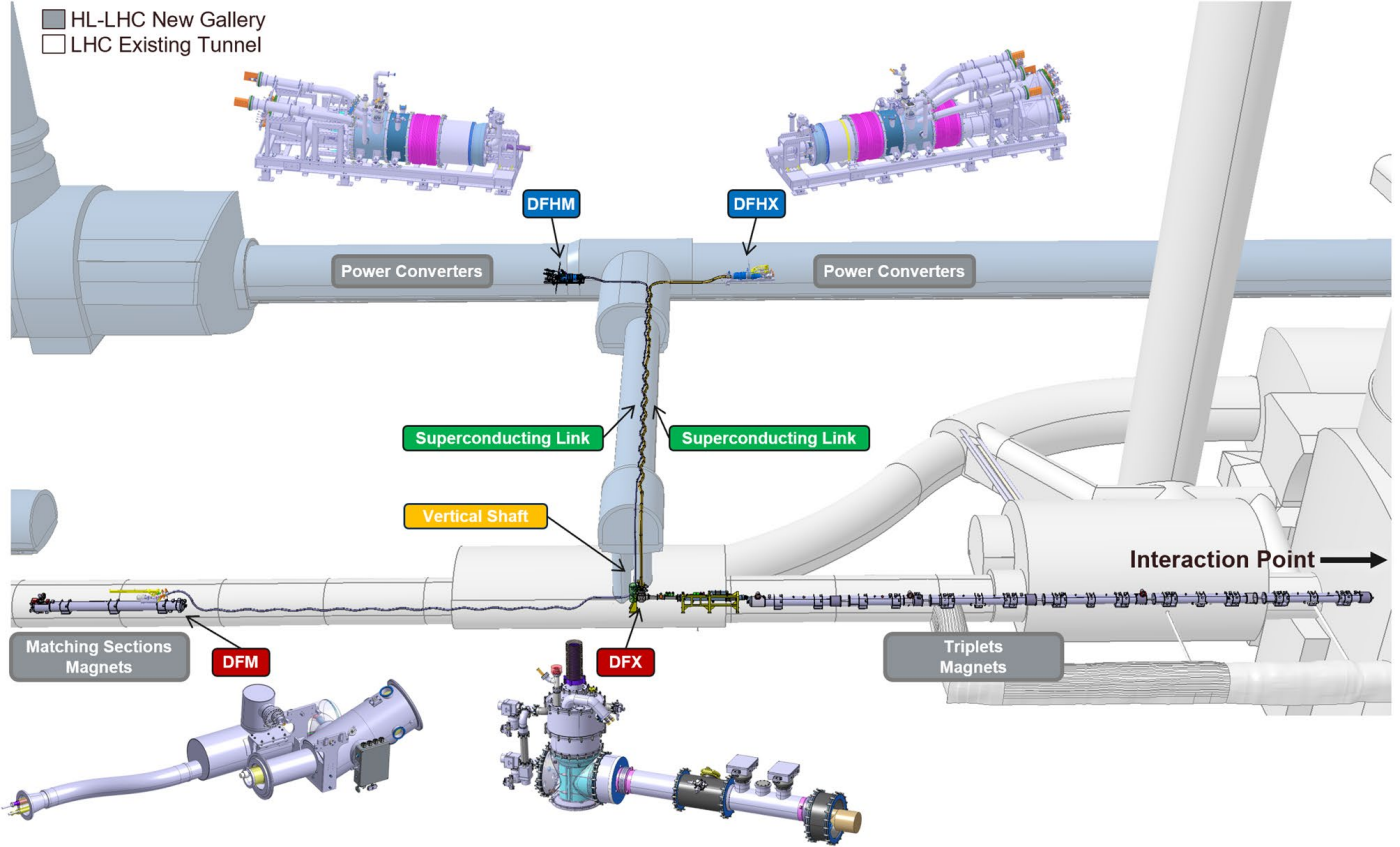
Schematic view of the LHC underground with the LHC existing tunnel and the HL-LHC new gallery where the Cold Powering Systems will be installed. The two types of systems that will be installed right and left of ATLAS and CMS experiments are shown in their final location. Magnified pictures of the DFHX, DFHM, DFX and DFM are also shown.

**Fig. 2:**
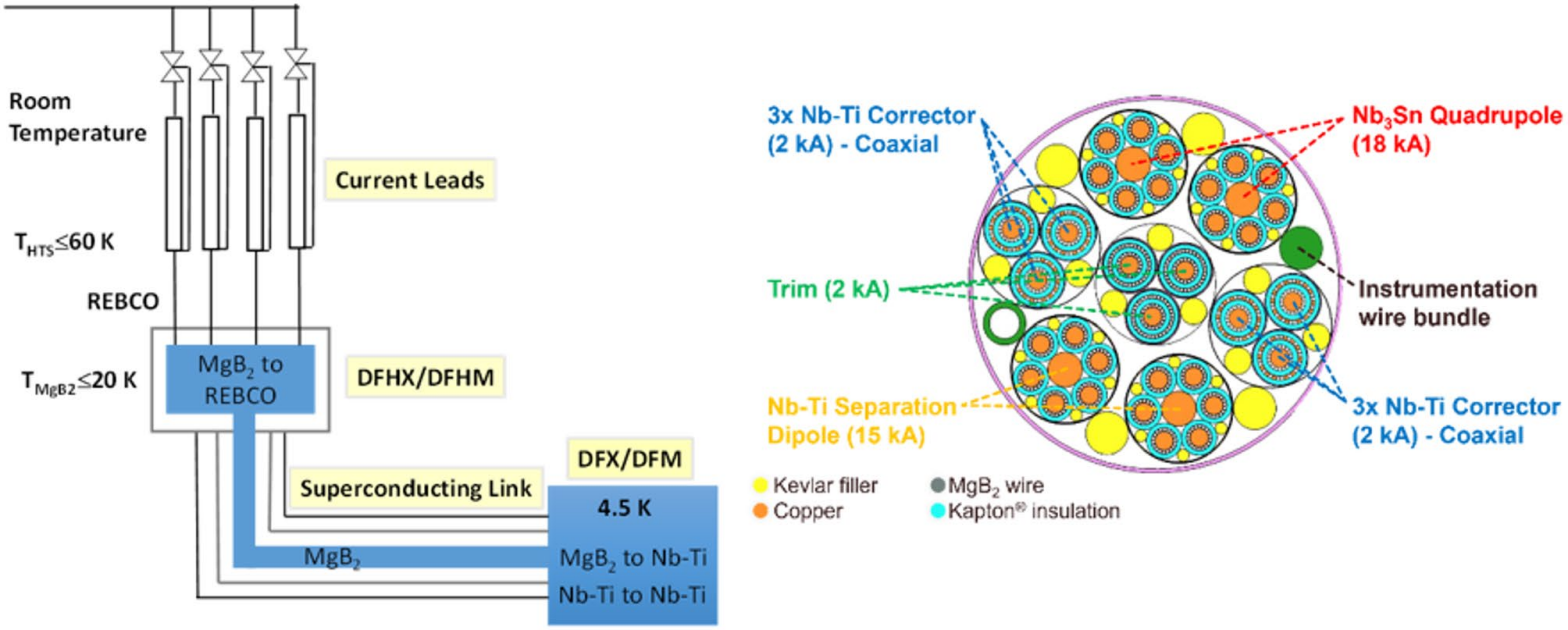
Schematic layout of a Cold Powering System (left). Nb-Ti cables are in a saturated liquid helium bath at 4.5 K, MgB_2_ cables operate between 4.5 K and about 20 K, and REBCO cables operate between 20 K and about 60 K. Layout of the |117| kA MgB_2_ cable assembly of the Superconducting Link for the HL-LHC Triplets (right). There are in total nineteen cables: two 18 kA cables for the Nb_3_Sn quadrupole magnets, two 15 kA cables for the Nb-Ti Separation Dipole magnet (rated at 18 kA), three 2 kA cables for the Trim circuit (rated at up to 7 kA), and six coaxial 2 kA cables, i.e. twelve 2 kA polarities. The maximum field experienced by the superconductor is 1 T. The total external diameter is about 90 mm. The weight is about 23 kg/m.

To enhance the accelerator’s availability and efficiency, the power converters for the HL-LHC magnets will be installed in newly excavated galleries, located eight meters above and separated from the LHC existing tunnel. This will facilitate access of personnel for maintenance and operational interventions that will take place in radiation free areas. The Superconducting Links will transfer the current from the power converters to the HL-LHC magnets. To interconnect the two different tunnel levels, they will pass through a purposely excavated, eight-meter-high, vertical shaft (Fig. 1). A Superconducting Link consists of a flexible cryostat, made of two multi-layer-insulated (MLI) concentric corrugated pipes, and a multiplicity of electrically insulated MgB_2_ cables in the inner pipe. These cables, which are grouped and twisted together to form a compact multi-cable assembly, feed the HL-LHC magnet circuits of the HL-LHC Triplets and Matching Sections. A Superconducting Link is connected at its warmer (∼ 20 K) end to a cryostat (Distribution Feedbox at Higher temperature for the Triplets, DFHX, or Distribution Feedbox at Higher temperature for the Matching Sections, DFHM) that contains the High Temperature Superconducting (HTS) Rare-Earth-Barium-Copper-Oxide (REBCO) based current leads providing the electrical transfer to room temperature, and at its colder (4.5 K) end to a cryostat (Distribution Feedbox for the Triplets, DFX, or Distribution Feedbox for the Matching Sections, DFM) that contains the Niobium Titanium (Nb-Ti) cables going to the magnets (Figs. 1 and 2, left). The system of the Superconducting Link connected to a cryostat at each end is called Cold Powering System. A Cold Powering System provides the electrical transfer from room temperature to the liquid helium environment of the magnets.

In total eight Cold Powering Systems of two different types are needed for HL-LHC: four for the powering of the magnets in the Triplets and four for the powering of the magnets in the Matching Sections. Two systems of different type will be located right and left of LHC Point 1 and Point 5 that house the ATLAS and CMS experiments. The Cold Powering Systems for the Triplets incorporate Superconducting Links that are 74.5 m long. They contain nineteen MgB_2_ cables (Fig. 2, right) rated at DC currents of 18 kA, 7 kA and 2 kA, and an equivalent number of HTS current leads and Nb-Ti cables. The MgB_2_ multi-cable assembly includes Kevlar^®^ fillers, which also strengthen the mechanical behaviour under tension. It has a diameter of about 90 mm and it can carry a total DC current of up to |117| kA, reaching a peak field in the conductor of about 1 T. The Cold Powering Systems for the Matching Sections incorporate Superconducting Links that are 120 m long. They require ten MgB_2_ cables, and an equivalent number of HTS current leads and Nb-Ti cables rated at currents of 18 kA and 0.6 kA. They can transfer a total DC current of up to |40| kA via a multi-cable assembly that has a diameter of about 60 mm.

The Superconducting Link will be transported and lowered in the LHC underground wound onto a spool with attached the DFHX or DFHM cryostat. The transport involves passing this system through the LHC shaft and maneuvering it in the underground while respecting the tunnel’s dimensions at different locations. These requirements have determined the maximum volume that a Superconducting Link and the corresponding DFH of DFHM cryostat can take up when arranged in the final configuration for transport. A compact design for the DFHX and DFHM has been developed. Additionally, the mechanical characteristics of the Superconducting Link have led to the establishment of its minimum bending radius of 2 m. This radius has been used as a boundary condition for underground installation: once the DFHX or DFHM have been placed at their final location in the new gallery (see Fig. 1), the Superconducting Link is unspooled and arranged in ducts on the floor or on wall-mounted supporting frames with a wavy configuration that adheres to the minimum bending radius. When the Superconducting Link moves away from the DFHX or DFHM, it travers the underground gallery, which is about 50 m long and perpendicular to the LHC tunnel (see Fig. 1) and then enters the eight-meter-high vertical shaft that leads it into the existing LHC tunnel. The DFX and the DFM are installed after unspooling in the underground. The DFX is located just beneath the shaft, while the DFM is approximately 50 m distant from the shaft within the LHC tunnel.

Following extensive research and development (R&D) as well as validation of individual components^3,4^, a Cold Powering System of the type needed for the HL-LHC Triplets has been constructed and extensively tested at CERN. This paper reports on the outcome of the room temperature, cryogenic, electrical and mechanical measurements performed on the first ever built 100 kA class MgB_2_ and REBCO system.

## Methods

### Test bench

**Fig. 3:**
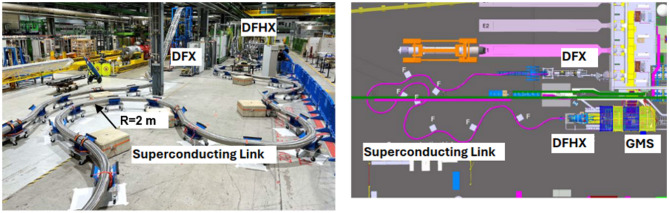
Cold Powering System in the test configuration in the SM-18 (left). The minimum bending radius (R) of the Superconducting Link is 2 m. Layout of Cold Powering System in the SM-18 (right). F are fix points.

**Fig. 4:**
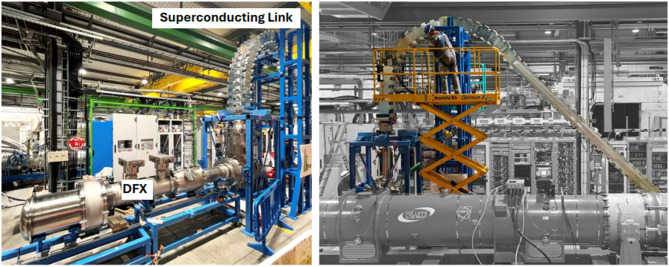
Vertical path of the Superconducting Link and DFX. The L-shape of the DFX cryostat is due to the presence of a horizontal part, with the Nb-Ti cables, and a vertical part where the MgB_2_ cables are connected to the Nb-Ti cables.

**Fig. 5:**
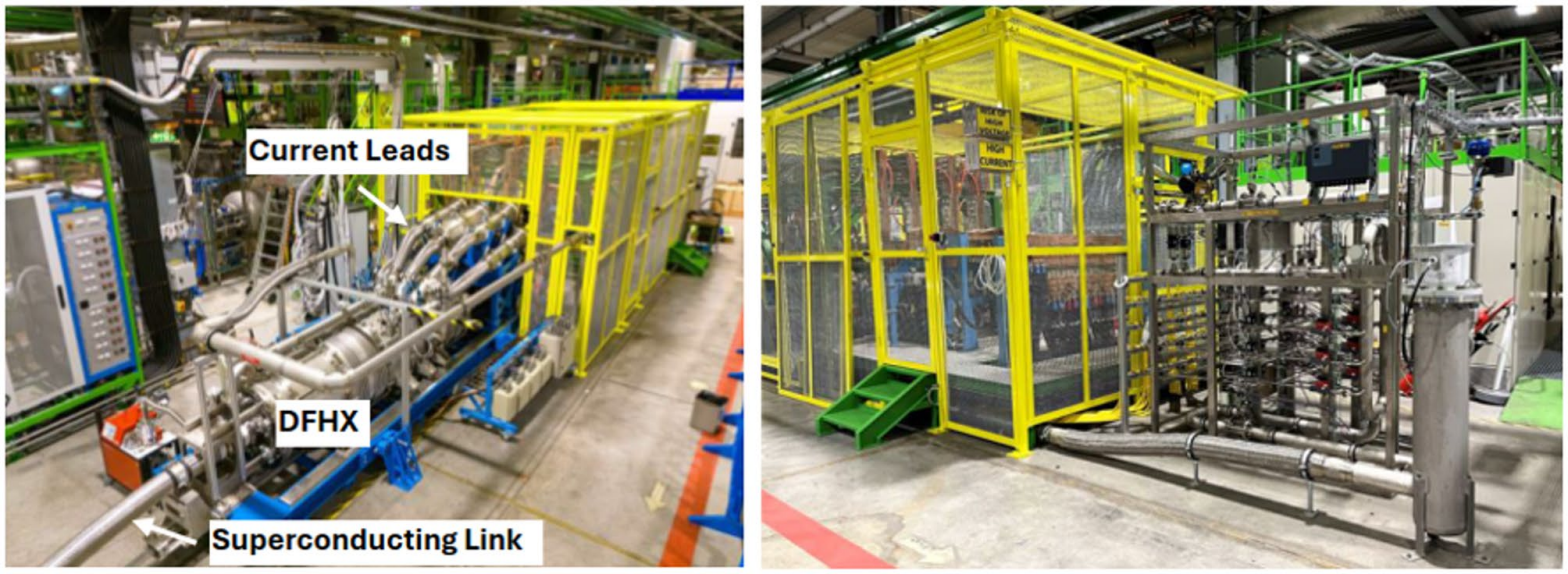
DFHX and room temperature cables inside the IP2X yellow cage. Gas management system in front of the IP2X yellow cage with the room temperature cables distributed and connected to the current leads. It contains helium valves and helium gas recovery lines.

The Cold Powering System was installed and tested at CERN in the SM-18 (Superconducting Magnet Test Facility). The Superconducting Link has a snaked geometry on the ground (Fig. 3): while this geometry does not reproduce the final configuration in the LHC underground, it meets the requirements of fitting the full length of 74.5 m within the available test area, coping with the thermal contraction and expansion of the system during cool-down and warm-up, and respecting the minimum bending radius of 2 m. To simulate the vertical path in the underground, where the Superconducting Link passes through the shaft connecting the HL-LHC new gallery to the LHC tunnel, a cable chain has been installed: it supports the Superconducting Link on the climb that leads to a vertical descent of about 2.5 m (Fig. 4). At the end of the descent, the DFX cryostat receives the MgB_2_ cables that are electrically connected in a vertical configuration to Nb-Ti cables. At the other side of the Superconducting Link, the MgB_2_ cables are distributed inside the DFHX cryostat and are connected to the REBCO cables that are part of the HTS current leads. Each REBCO cable is connected to the resistive part of a current lead. The DFHX hosts, in a compact volume (∼ 5.5 m length, ∼ 1 m external diameter), the nineteen electrical joints (splices) between the MgB_2_ and the REBCO cables and the nineteen HTS current leads. For the benefit of compactness, the current leads are not aligned vertically, one after the other, in the DFHX like in the LHC (Fig. 1 in^5^). They are instead grouped at two locations of the DFHX and are almost horizontal to the ground – the inclination ranges from about 6 to 15 degrees. Use of helium gas instead of liquid cryogen enables robust operation of the current leads in this configuration (Fig. 5, left).

**Fig. 6:**
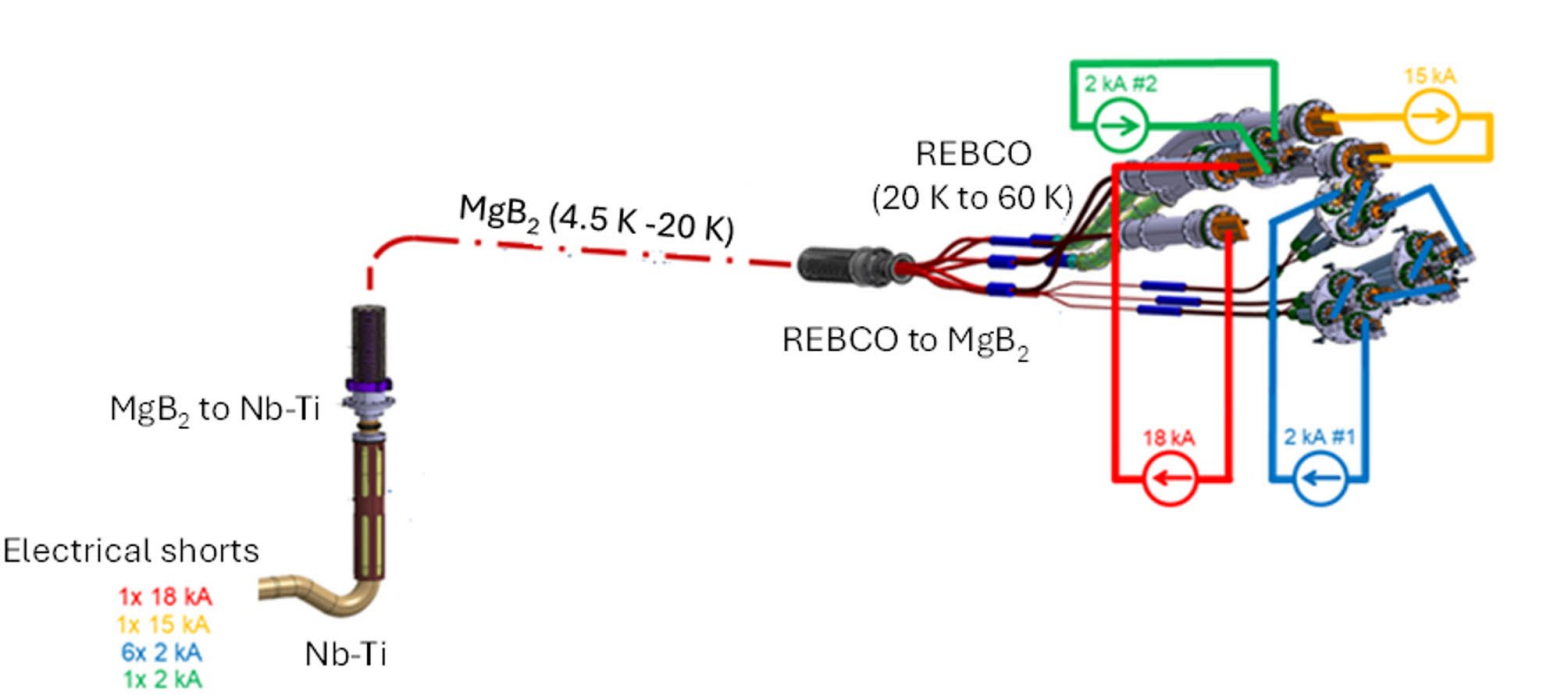
Powering layout adopted for the tests. Four power converters (2 kA, 2 kA, 15 kA and 18 kA) are available. The number of electrical shorts inside the DFX at 4.5 K, between Nb-Ti cables, is also reported.

Four power converters are available for the electrical tests: one 18 kA, one 15 kA and two 2 kA. Powering of an 18 kA circuit (Nb_3_Sn Quadrupole circuit in Table 1), of a 15 kA circuit (Nb-Ti Separation Dipole circuit in Table 1), and of two 2 kA circuits (Trim and Nb-Ti Corrector circuits in Table 1, the latter all powered in series) is therefore possible. The powering scheme is reported in Fig. 6. The routing of the room temperature water cooled 18 kA and 15 kA cables and of the air cooled 2 kA cables as well as their connection to the current leads are inside an Ingress Protection (IP) 2X rated cage, in front of the DFHX, that reproduces the final configuration in the HL-LHC gallery (Fig. 5). The cage ensures protection against solid objects according to the IEC 60529 standard.

A dedicated cryogenic line transfers liquid helium from an available source, in the SM-18, to the DFX cryostat: a mix of liquid and gaseous helium is injected at 1.3 bara inside the DFX. Liquid helium fills the DFX cryostat and covers the electrical splices between MgB_2_ and Nb-Ti. Two proportional-integral-derivative (PID) controlled 100 W electrical heaters inside the DFX vaporize the liquid helium to provide the gaseous mass flow rate required to cool the system. The gas flows along the Superconducting Link and reaches the DFHX where it is distributed among the nineteen MgB_2_ to REBCO splices and the nineteen current leads before being recovered at room temperature.

Recovery of helium gas on the DFHX side is managed by a purpose-built Gas Management System (GMS, see Fig. 3), which groups the helium gas return piping at room temperature and the valves used for the control of the flow through the current leads (Fig. 5). In the DFHX there is also a cryogenic by-pass line that recovers the excess of cold gas and warms it up to room temperature via a 15 kW electric heater.

## Operating conditions

The number and type of HL-LHC circuits and the number of cables and current leads in the Cold powering System are listed in Table 1. The current at which each circuit has been tested (test current), corresponding to the maximum current of the power converter available in the SM-18, the current at which each circuit will operate in the LHC (nominal current), and the design current (maximum current delivered by the power converter in the LHC) are also reported The design current provides a margin of at least 10% with respect to the nominal current. The total current is the sum of the absolute value of the current transferred by each polarity in the Cold Powering System.

**Table 1:** Number of HL-LHC circuits fed by the Cold Powering System, number of cables (MgB_2_ or REBCO) and current leads per circuit type. Nominal currents during operation in the HL-LHC, test currents and design currents of the circuits are reported.

	Number of Circuits	Number of Cables/Current Leads	Nominal Current (kA)	Test Current (kA)	Design Current (kA)
Nb_3_Sn Quadrupole	1	2	16.23	18	18
Nb-Ti Separation Dipole	1	2	12.11	15	18
Nb-Ti Trim	1	3*	2	2	7**
Nb-Ti Corrector	2	4	1.74	2	2
Nb-Ti Corrector	2	4	1.34	2	2
Nb-Ti Corrector	2	4	1.59	2	2
Total	9	19	81.36	94***	117****

* The Trim circuit adjusts the current of the Nb_3_Sn Quadrupole circuit. It consists of three MgB_2_ cables and three current leads.

** The MgB_2_ and REBCO cables of the Trim circuit can transfer a current of up to 7 kA to cope with transients in the magnet circuit.

*** Two cables/current leads of the Nb-Ti Trim circuit can be tested simultaneously.

****Total DC design current of the Superconducting Link.

The Cold Powering System should by design operate with a helium mass flow rate of not more than 5.5 g/s – when all circuits are operated at their nominal current. It must also be able to generate and transfer, in transient conditions, up to 10 g/s of helium gas produced in the DFX. The Superconducting Link operates in a temperature range from 4.5 K, in the DFX, to about 20 K, in the DFHX. The cryogenics of the Cold Powering System shall guarantee that: the Nb-Ti cables and their splices to the MgB_2_ cables are submersed in a saturated liquid helium bath inside the DFX; the MgB_2_ cables in the Superconducting Link operate at not more than 20 K; the REBCO cables in the current leads never exceed 60 K. These boundary conditions are by design defined as nominal cryogenic conditions.

The static heat load of the Superconducting Link was specified and measured, in a previous test campaign at CERN, to be 1.6 ± 0.5 W/m^6^. The helium mass flow through each current lead is optimized for operation at the design current in Table 1 and shall be of the order of 0.055 g/(s⋅kA).

The DFX internal vessel has a “fountain” configuration: there are two concentric volumes of saturated liquid helium. Helium is injected in the central volume, where the Nb-Ti cables and their splices to the MgB_2_ cables are located, and overflows into the outer volume. The outer volume contains an electrical heater, which provides the helium mass flow rate required for the cooling of the system, and the level gauge used for liquid helium level control. This design enables meeting the requirement, imposed by cryogenic operating conditions, of maintaining the MgB_2_ to Nb-Ti splices immersed in liquid helium during at least 10 minutes after an accidental stop of helium supply.

The helium gas produced in the DFX warms up, while absorbing the static heat load of the Superconducting Link cryostat, from 4.5 K up to a maximum temperature of 20 K at the location of the splices between the MgB_2_ and the REBCO cables. It then cools the nineteen current leads, at the exit of which it is recovered at room temperature. The temperature of the MgB_2_ to REBCO splices (T_MgB2_ ≤ 20 K) and of the warm termination of the REBCO cables (T_HTS_ ≤ 60 K) are monitored and the temperatures T_HTS_ are controlled. Room temperature valves, one per current lead, control the helium mass flow rate passing through each lead so that T_HTS_ stays at not more than 60 K. By design, T_HTS_ is expected to be in the range 50–60 K.

Neither the Superconducting Link nor the DFX and the DFHX cryostats include a thermal shield. Insulation from thermal radiation from room temperature to the cryogenic environment is provided exclusively by 30 to 40 multi-layer insulation blankets located around the cold inner part of the cryostats that contains liquid or gaseous helium. This simplified design is made possible by the use of superconducting materials operated at temperatures higher than liquid helium.

### Electrical requirements and instrumentation

The Cold Powering System includes high-current Nb-Ti, MgB_2_ and REBCO cables. The splices between MgB_2_ and REBCO are in helium gas in the DFHX, and those between MgB_2_ and Nb-Ti are in liquid helium in the DFX (see Fig. 2). While the splices are not required to be superconducting, low resistance is necessary to avoid local thermal run-away and ensure efficient cryogenic cooling of the system with a minimum mass flow rate.

Boundary conditions for the design were: electrical insulation among circuits and to ground of 2.3 kV when the system is in nominal cryogenic conditions; controlled cross talk among circuits, i.e. fast discharges generated by the resistive transition of the Nb-Ti Corrector magnets (see Table 1) should not trigger by electro-magnetic coupling the resistive transition of the other circuits. Circuits were therefore powered both individually and simultaneously with different ramp rates. Ramp rates ranging from 5 A/s to 100 A/s were tested.

**Fig. 7:**
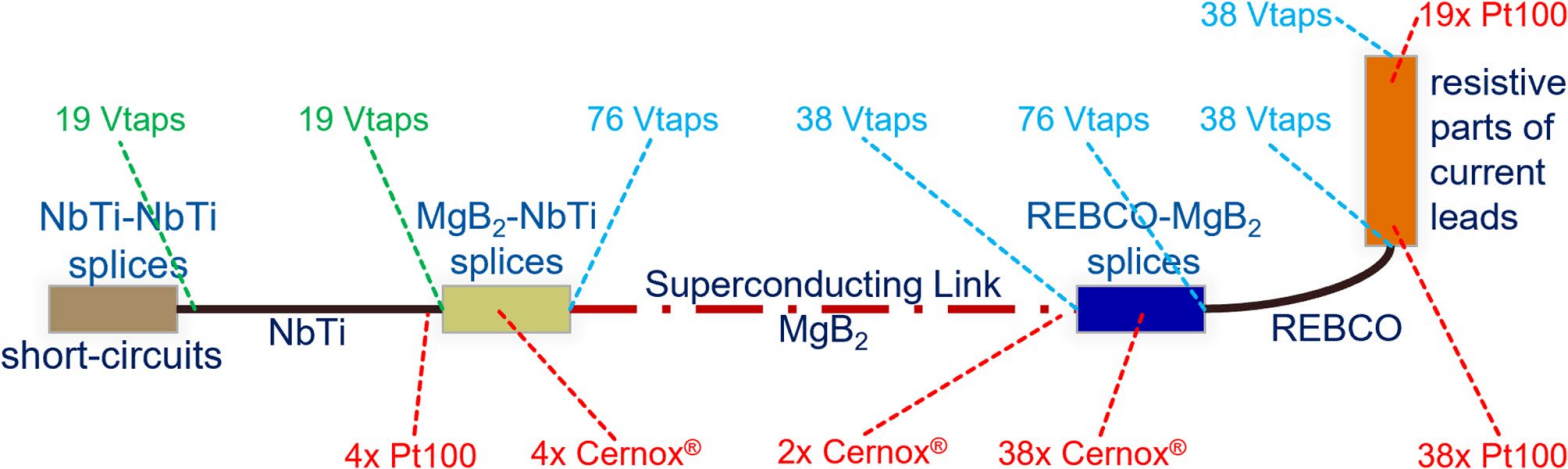
Location of voltage taps (VTaps) and temperature sensors (Cernox^®^ and PT100) in each circuit. VTaps used for protection functionalities are in light blue while Vtaps used for monitoring functionalities are in green. The total number of voltage taps (304) and temperature sensors (105) for all circuits is also reported.

If quench protection thresholds, i.e. the maximum allowed voltage drops along the superconducting parts of the system, are accidentally exceeded or nominal cryogenic conditions are lost, a discharge of the concerned circuit(s) is triggered. The temperature sensors and voltage taps required for monitoring and protection are incorporated in the system. All voltage taps used for quench protection are doubled for redundancy. Altogether, the system includes 304 voltage taps, 266 for protection and 38 for monitoring functionalities. An overview of the instrumentation is given in Fig. 7.

Data acquisition and quench detection are provided by twenty crates of the universal quench detection system (uQDS^7^). The crates are equipped with 16 inputs each for a total of 320 channels. They are arranged in two redundant groups of ten crates. Each crate and its redundant twin monitor and protect the two polarities of a circuit and measure with two analog inputs the current signals stemming from two independent direct current transducers (DCCT) of the circuit. Protection thresholds are: 100 mV for the resistive part of each current lead, 5 mV for each REBCO cable, and 20 mV for each MgB_2_ cable. Splices are also individually protected with voltage thresholds of 20 mV, for the MgB_2_ to Nb-Ti splices, and 5 mV, for the MgB_2_ to REBCO splices. The discrimination time of the quench detection system is 100 ms for the resistive part of the current leads and 20 ms for the REBCO and MgB_2_ cables as well as for the splices. At low currents, defined as ≤ 10% of the design current of a circuit (see Table 1), the quench detection thresholds are increased by a factor ten to compensate for the higher ripple of the power converters in those current ranges.

The Cold Powering System includes 105 temperature sensors (Fig. 7): sixty-one Platinum Resistance Temperature Detectors (RTD) Pt100 and forty-four Cernox^®^. The Pt100 sensors are located at the warm end of the REBCO cables (T_HTS_ in Fig. 2) where they are connected to the resistive part of the current lead – one sensor and a redundant twin per cable. They are used for the control of the flow through each current lead and their set point is 50–60 K. The Cernox^®^ are located on the MgB_2_ to REBCO splices (T_MgB2_in Fig. 2) – one sensor and a redundant twin per splice. Both sensors are part of the interlock chain of the circuits: a power abort of a circuit (50 A/s to 100 A/s discharge, depending on the circuit) is triggered if a sensor exceeds by 5 K the nominal operating value, i.e. if the temperature of a MgB_2_ to REBCO splice reaches 25 K or if the temperature of the REBCO reaches 55–65 K. In addition, one Pt100 sensor is incorporated in the room temperature terminal of each current lead. The power abort of the concerned circuit is triggered if it exceeds 320 K or if is lower than 275 K. Below 275 K, the gas flow through the concerned current lead is also interlocked. This ensures that neither overheating nor overcooling of the current leads can take place in the system.

The electrical tests aim at qualifying each circuit of the system. The test bench enables powering the circuits in Table 1 according to the following layout: the 18 kA circuit (Nb_3_Sn Quadrupole) is powered individually (the two polarities are electrically shorted inside the DFX via a Nb-Ti to Nb-Ti splice); the 15 kA circuit (Nb-Ti Separation Dipole) is powered individually (the two polarities are electrically shorted inside the DFX via a Nb-Ti to Nb-Ti splice); the 2 kA circuit (Nb-Ti Trim) is powered individually (two polarities plus one spare cable are electrically shorted inside the DFX via Nb-Ti to Nb-Ti splices and can be powered in pairs by changing the connections at the room terminal of the current leads); the 2 kA circuits of the corrector magnets (Nb-Ti Correctors) are powered all in series (the twelve polarities are electrically shorted inside the DFX via six Nb-Ti to Nb-Ti splices and at room temperature at the level of the current leads terminal). The powering layout is reported in Fig. 6. The nineteen polarities – corresponding to nine circuits – could be powered individually via four power converters.

## Results

### Measured cryogenic performance

The test campaign started with a pressure test of the Cold Powering System at 4.6 bara, followed by a helium leak test. Both tests were successful, and the helium leak rate was measured to be better than the specified value (≤ 1.0 × 10^−8^ mbar·l·s ^−1^).

**Fig. 8:**
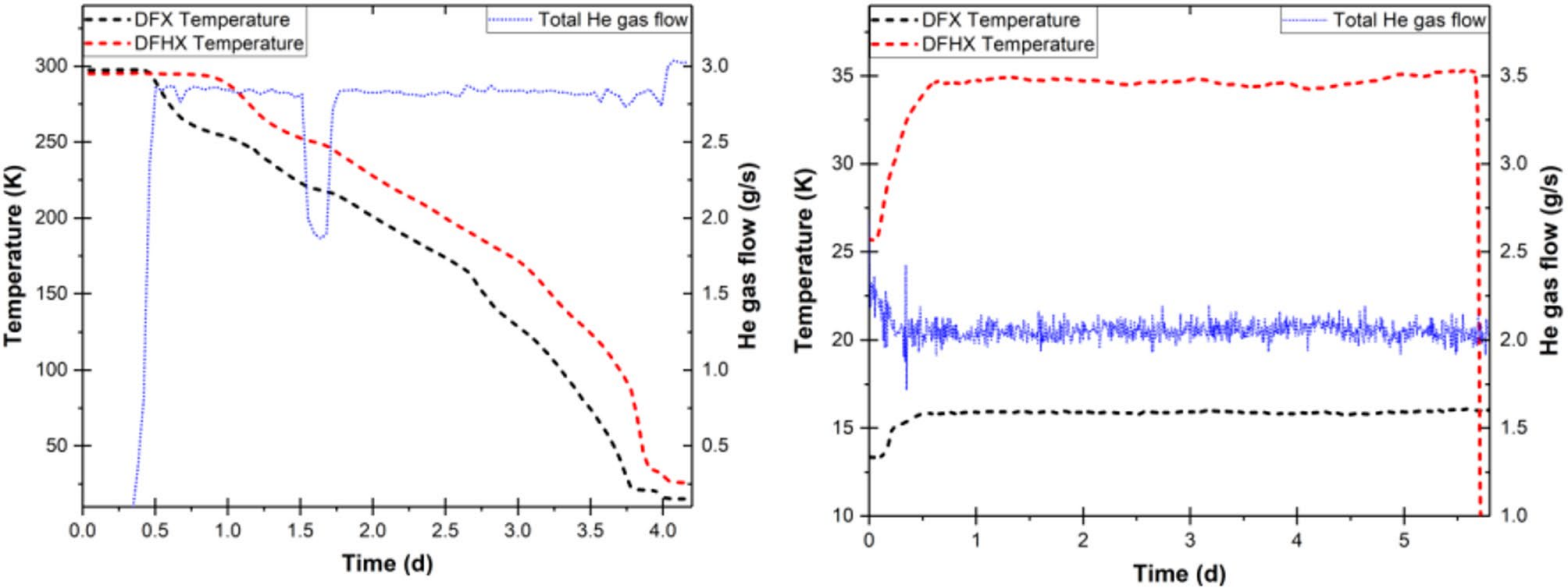
Cool-down of the Cold Powering System from room temperature to about 15 K inside the DFHX (left). The decrease in helium gas flow after about 1.5 days is due to a failure of the cryogenic control system. The flow was re-established after about 0.5 days with no consequences on the system. Stable cryogenic conditions maintained during about 5 days for the measurement of the thermal performance of the Superconducting Link (right).

The cool down of the system was performed with a helium gas mass flow rate of 2 to 3 g/s. The thermal gradient between the helium supplied in the DFX and the helium recovered in the DFHX was limited to 50 K during the transient from room temperature to 160 K, and then increased to 70 K until the helium supplied in the DFX reached 15 K. The temperature gradient was increased from 50 K to 70 K when the thermal contraction and movements in the vertical part of the Superconducting Link, close to the DFX, had taken place. This phase took about 3.5 days. Nominal cryogenic conditions – with liquid helium inside the DFX – were then reached in about eight hours (Fig. 8, left). This cool-down procedure will be adopted for cool-down during operation in the LHC.

For dealing with the thermal contractions, the Superconducting Link was installed on the ground with a wavy shape that enables movements during cool-down/warm-up as well as during the pumping of the thermal insulating vacuum inside the flexible cryostat. The waves are fixed to ground at some locations and free to move thanks to dedicated guide rollers elsewhere (see Fig. 3). The amplitude and geometry of the waves were measured before and after vacuum pumping and cool down. Vacuum and cool down generated a maximum radial displacement of the peak of the free waves of 30 mm. After warm-up the Superconducting Link recovered its initial geometry with a maximum deviation of 10 mm due to stick-slip effect on the ground.

During the electrical tests, the system was powered from 0 kA to |94| kA (see Table 1), with |94| kA DC current maintained up to eight hours. The helium mass flow rate required to cool the full system (DFH, Superconducting Link, DFHX and current leads) when operated at |94| kA was measured to be 4.9 g/s ± 0.1 g/s. Measured temperatures met nominal cryogenic conditions and no temperature drifts could be detected in the system. The total pressure drop of the helium was measured to be < 30 mbar during cool-down and < 10 mbar in nominal cryogenic conditions. This is well within the maximum acceptable value of 50 mbar.

At zero current, a helium mass flow rate of 3.5 g/s was sufficient for cooling the system and maintaining it at nominal cryogenic conditions. In this operating mode, a flow of 5 g/s was generated in the DFX, and 1.5 g/s were extracted at the level of the cryogenic by-pass line in the DFHX. This is done to ensure a buffer of helium gas close to the current leads, to cope with a transient flow increase due to powering, and to guarantee a precise control of the liquid helium level (± 1 cm) inside the DFX, which implies continuous operation of the electrical heater in the DFX outer volume. A test with a reduced helium mass flow rate was also performed. The goal of this test was to define a cryogenically economic configuration that can be adopted during long periods with no liquid helium and no current in the system, i.e. during a stand-by mode usually corresponding to maintenance interventions in the accelerator. A mass flow of 3 g/s with helium gas entering the DFX at 20 K was able to maintain the REBCO in the current leads (T_HTS_) at a temperature ≤ 100 K.

**Fig. 9:**
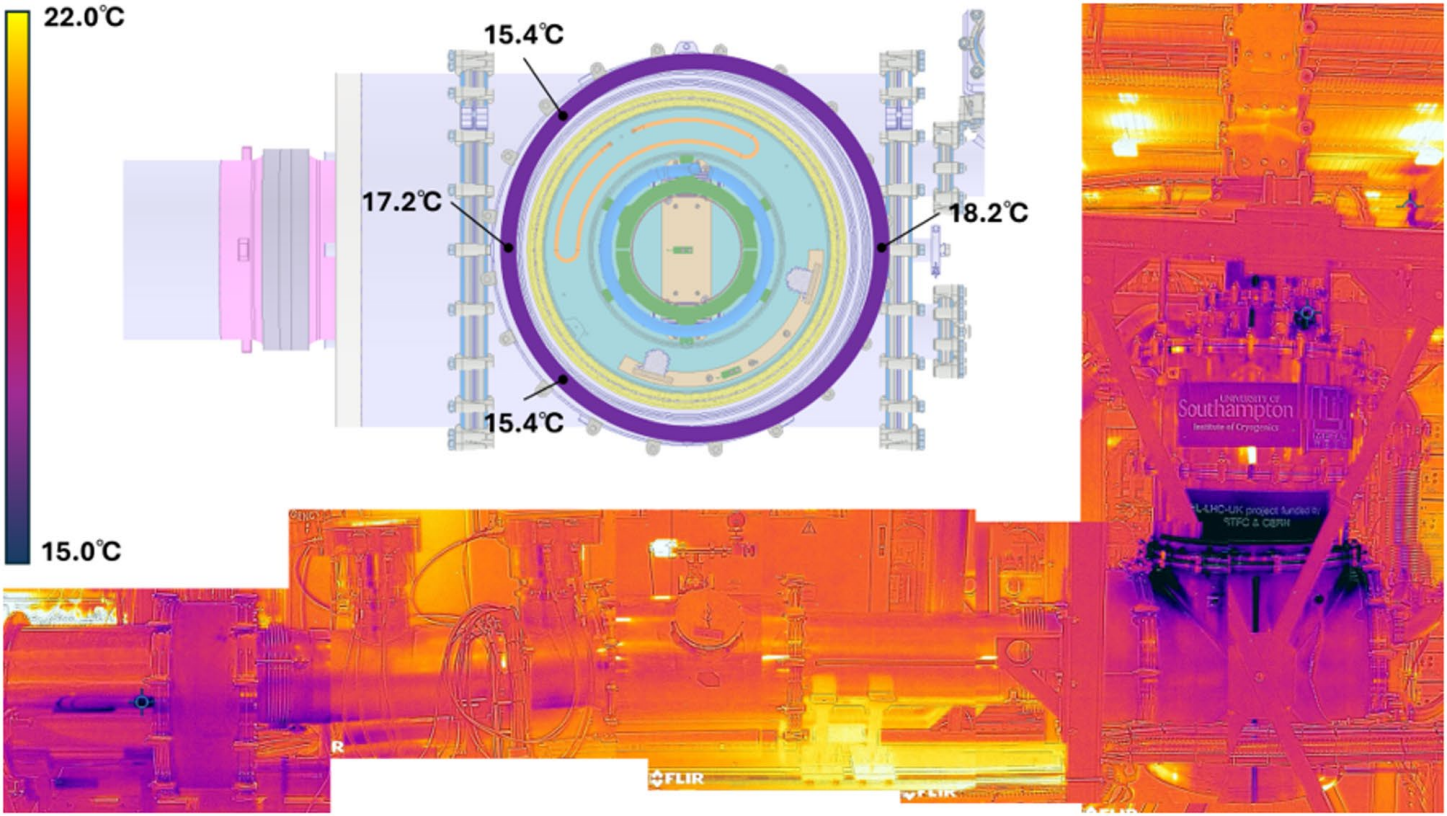
Thermal mapping of DFX cryostat in nominal cryogenic conditions and during powering.

The thermal performance of the Superconducting Link was quantified during five days of steady state cryogenic operation with 2 g/s of helium gas flowing through the system (Fig. 8, right) and an inlet temperature of 15.8 K. The measured heat load is 2.0 W/m ± 0.5 W/m, in line with more precise measurements performed in the past^6^. A temperature mapping campaign with infrared camera enabled excluding presence of condensation or cold areas at any location along the external wall of the Superconducting Link cryostat and anywhere else in the system. For the assessment of the cryogenic performance of the DFX cryostat, two boil-off tests (measurement of rate of decrease of the liquid helium level with no helium supply) were performed as well as a temperature mapping campaign of the external wall of the cryostat with a thermal imaging system. No condensation could be observed at any location, but some colder areas (15.4 °C to 19.1 °C) were identified (Fig. 9). The total static heat load was quantified to be 67 W, 55 W of which deposited into the liquid helium volume, at 4.5 K, and the remaining 12 W deposited into the helium gaseous volume above the liquid. This figure presents an extra 30 W with respect to the estimations^8^. It is considered that 10 W are due to a thermal shortcut through compacted MLI superinsulation blanket and 20 W are due to a non-optimised installation of MLI blankets. While optimal cryostat design is always a compromise between minimising heat inleak and adequate mechanical robustness, the mechanical design of the DFX cryostat had two unique challenges: (a) the inner helium vessel of the DFX cryostat has a design pressure of 3.5 bara (tested at 5 bara for European Conformity (CE) marking as a Category III Pressure Equipment) and is required to withstand high bending moment due to the L-shape (see Fig. 4) needed for keeping the MgB_2_ to Nb-Ti splices in liquid helium and for transitioning the Superconducting Link from a vertical to an horizontal configuration; (b) the mechanical support for the 600 mm diameter vertical section of the inner vessel is restricted by the space available in the LHC tunnel to a short length of 300 mm between 4.2 K and 300 K to the detriment of thermal conduction. The test results fully validated the innovative structural design. For the remaining cryostats, the MLI installation will be further optimised with respect to the layout used in this test.

During the powering tests, neither condensation nor cold areas could be observed on the DFHX external envelope or on the current leads. For the current leads, the temperature of the REBCO was set between 50 K and 60 K leading to the definition of the optimized operating temperatures: T_HTS_ = 50 K for the 18 kA and for the 15 kA current leads, and T_HTS_ = 50–60 K for 2 kA current leads. The mass flow requirements of the current leads were measured to be: 0.81 ± 0.03 g/s for the 18 kA, 0.68 ± 0.01 g/s for the 15 kA, 111 ± 8 mg/s to 142 ± 8 mg/s for the 2 kA.

The capability of generating and operating with a mass flow rate of 10 g/s was demonstrated. Two boil-off tests were performed, and it was shown that that such a flow can be produced by evaporation of liquid helium inside the DFX – with the level of liquid helium maintained within specification. It was also demonstrated that the MgB_2_ to REBCO splices remain submersed in liquid helium if the supply is interrupted for 10 min.

The tests demonstrated the cryogenic efficiency of the system that was operated in DC mode with a helium mass flow rate of 4.9 ± 0.1 g/s at |94| kA and in nominal cryogenic conditions. The measured flow rate is well within the maximum value (5.5 g/s) defined acceptable during the design phase. Stability and robustness of the cryogenic control were also proven.

### High voltage electrical insulation tests

Electrical insulation of the superconducting cables is provided by multi-layer wrapping of polyimide tape, while splices are insulated via machined glass-fibre-reinforced-plastic (G-10) and ULTEM™ parts. Insulators guarantee a minimum helium path in between non-insulated parts of 30 mm. A high voltage insulation test consists in measuring the electrical insulation between each polarity of a circuit and all the others and between each polarity of a circuit and the ground. The most critical part of the system with respect to the electrical insulation is the compact multi-cable assembly of the Superconducting Link.

The electrical insulation of the MgB_2_ cables was tested at up to 15 kV, at room temperature and in air, after production of the multi-cable assembly. After completion of the assembly of the Cold Powering System the following tests were performed: 5 kV at room temperature and in air before cool-down, 2.3 kV with the system in nominal cryogenic conditions, and 1.1 kV with the system filled with helium gas at 1.1 ± 0.015 bara and at room temperature after the completion of the powering tests and warm-up. During operation in the LHC, the high voltage tests will also be performed at 2.3 kV in nominal cryogenic conditions and at 1.1 kV at room temperature with helium gas at 1.1 bara inside the system. The maximum leakage current for each circuit is specified to be 10 µA during the voltage plateaus (≥ 180 s) and 100 µA during the voltage ramps (50 V/s). The system passed successfully all high voltage insulation tests. The maximum leakage current measured during the 2.3 kV test in nominal cryogenic conditions was 104.0 nA – about 100 times lower than the maximum specified. Instrumentation signals, associated feedthroughs and temperature sensors all successfully passed the high voltage tests.

### Measured electrical performance

**Fig. 10:**
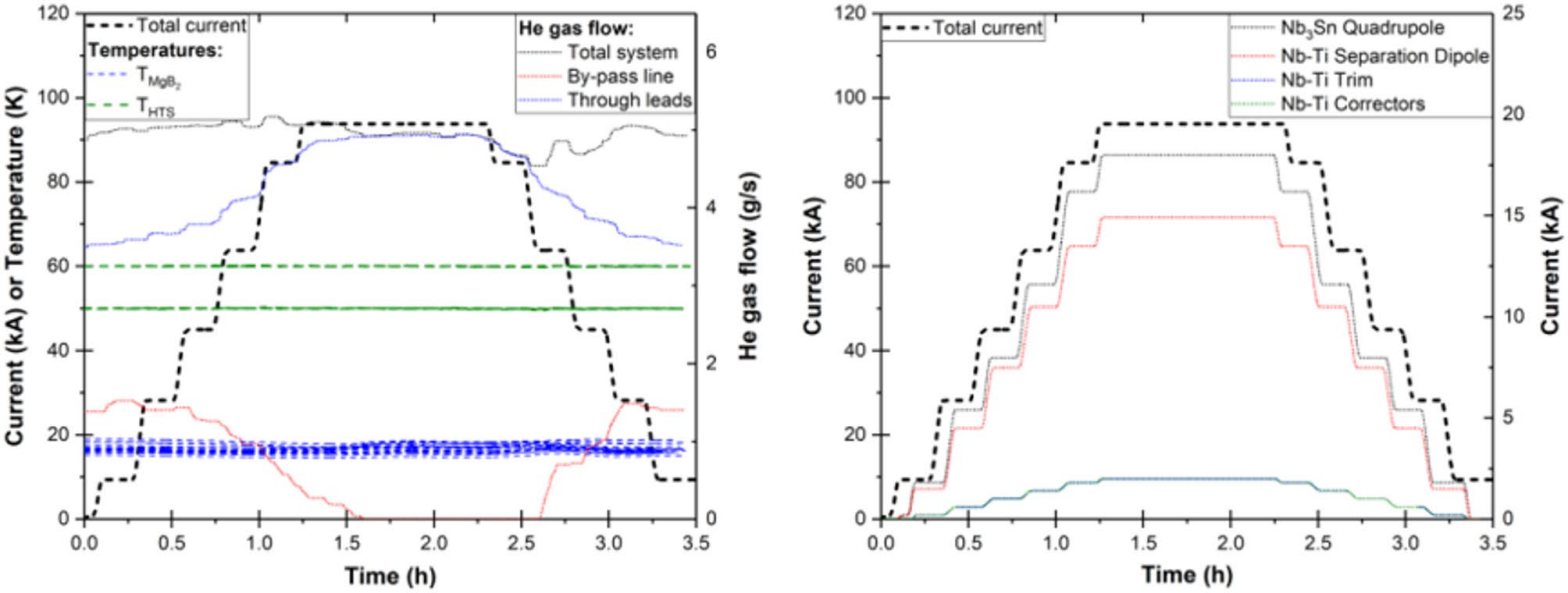
One-hour steady state test current in all circuits (2 kA in the Trim circuit, 2 kA in the Corrector circuits, 15 kA in the Separation Dipole circuit and 18 kA in the Quadrupole circuit). Ramp rates ranging from 5 A/s to 20 A/s were adopted. The mass flow rate through the system in nominal operating conditions is 4.9 ± 0.1 g/s. On the left the total current is reported. On the right the individual current in each circuit is reported.

The test sequence adopted for the powering tests can be summarized as follows:

- The nine electrical circuits were individually powered to their test current (18 kA, 15 kA, 2 kA and 2 kA, see Table 1). Nominal cryogenic conditions were ensured. The valves successfully controlled the helium mass flow through each current lead maintaining constant the T_HTS_ of each REBCO cable (Fig. 10). Each circuit was brought individually in six steps from zero current to the test current. Each step was held for about 10 minutes. Ramp rates from 5 A/s to 20 A/s were selected to obtain a synchronized current ramp in all circuits;

b) The test current was then maintained for one hour in all circuits (Fig. 10). For one hour, the system operated in nominal operating conditions with a total current of |94| kA, i.e. the maximum current that could be delivered by the four power converters, and a mass flow rate of 4.9 ± 0.1 g/s. Afterwards, each circuit was brought back to zero current with the same ramp rate and plateaus at lower currents;
c) All circuits were ramped simultaneously with synchronized ramp rates ranging from 20 A/s to 100 A/s both in steps and from zero to their test current. The test currents were maintained constant in all circuits during eight hours with stable cryogenic conditions. Individual and simultaneous powering of all circuits were successfully repeated after a complete thermal cycle of the Cold powering System: the system was warmed-up to room temperature, cooled-down again to nominal cryogenic conditions, and powering tests were repeated.

During all tests no resistive transitions took place in the superconducting parts (Nb-Ti, MgB_2_ and REBCO) of the circuits, no temperature or voltage drifts could be detected, voltage drops across resistive sections were within estimated values, temperatures and liquid helium level were stable and corresponding to those selected for nominal cryogenic conditions.

Validation of the electrical splices was an important qualification step. Both REBCO tapes and MgB_2_ wires contain high resistance metal in their matrix or substrate, Hastelloy^®^ and Monel^®^, respectively, and optimization of the splices required a significant R&D effort. REBCO to MgB_2_ splices are cooled by forced flow of helium gas at about 20 K inside the DFHX. They were measured to be in the range from 1.4 nΩ to 10.1 nΩ. MgB_2_ to Nb-Ti splices are in liquid helium at 4.5 K inside the DFX. They were measured to be in the range from 1.4 nΩ to 2.4 nΩ. The electrical splices between Nb-Ti cables, used to create the electrical shorts in liquid helium inside the DFX, were less than 1.5 nΩ. All measured values matched with the estimated ones (see Table 2). They did not change after hundreds of electrical cycles and one complete thermal cycle (from room temperature to nominal cryogenic conditions) of the Cold Powering System.

The evolution of the temperatures of the REBCO to MgB_2_ splices during the current cycles are shown in Fig. 10, left. The temperatures of all REBCO to MgB_2_ splices remained at the nominal value (< 20 K) and are independent of the current. No indication of heating of the splices, during any phase of the test could be identified: both measured resistances and measured temperatures of the splices were stable.

**Table 2:** Resistance of the splices measured and estimated via analytical calculations.

Test Current (kA)	REBCO to MgB_2_		MgB_2_ to Nb-Ti		Nb-Ti to Nb-Ti	
	R_splice_ Measured (nΩ)	R_splice_ Estimated (nΩ)	R_splice_ Measured (nΩ)	R_splice_ Estimated (nΩ)	R_splice_ Measured (nΩ)	R_splice_ Estimated (nΩ)
18	1.4 ± 0.1	≤ 2.2	1.4 ± 0.1	≤ 1.8	0.9 ± 0.1	≤ 2.0
15	1.7 ± 0.1		1.4 ± 0.3		0.9 ± 0.1	
2 Trim	4.3 ± 0.8	≤ 6.5	1.4 ± 0.2	≤ 3.5	1.2 ± 0.1	
2 Correctors	10.1 ± 1.1	≤ 13.0	2.4 ± 1.4	≤ 6.0	1.1 ± 0.3	

The mechanical design of the Cold Powering System relies on the flexibility of the Superconducting Link, which accommodates thermal contraction and expansion via its convoluted geometry. It also includes a fix point of the MgB_2_ cables at the location where they enter the DFHX. The highest force experienced by the MgB_2_ cable assembly occurs during its pulling inside the cryostat of the Superconducting Link – in the construction phase. This force is about 12 kN. The force on the MgB_2_ cable assembly when positioned vertically in the LHC shaft is about 1.8 kN. An extensive measurement campaign performed at an early stage of the project confirmed that such forces do not impact on the MgB_2_ performance. The tests performed on the Cold Powering System confirmed the robustness and reliability of its mechanical design.

### Electro-magnetic compatibility

Electro-magnetic cross talk among circuits, that could trigger the quench detection system, should be avoided during operation. More specifically, it is requested that in the case of quench of a corrector magnet in a 2 kA circuit, the resulting fast discharge of that circuit should not trip the quench protection of neither the Nb_3_Sn Quadrupole (18 kA) nor the Nb-Ti Separation Dipole (15 kA) circuits. Limitation of inductive couplings has therefore been addressed in the design of the Superconducting Link. This behaviour has been investigated by measuring the self-inductance of each circuit and the cross talk between circuits. Each circuit was powered with ramp rates of 50 A/s to 100 A/s and the voltage induced during the ramp in the other circuits was measured. The response of a circuit was found to be dependent on the relative position of the MgB_2_ cables in the Superconducting Link and on the routing/location of the associated voltage signals. Measured inductive couplings between circuits and the self-inductance of each circuit are reported in Table 3. The coaxial MgB_2_ cable layout of the 2 kA circuits results in a very small inductive couplings of 0.02–0.03 µH towards the other circuits.

A fast power abort in the HL-LHC configuration triggers a 6 kA/s discharge of the 2 kA corrector circuits. The measured maximum inductive coupling of 0.03 µH towards any other circuit implies induced voltages in the MgB_2_ cables of the other circuits of 0.2 mV, a value about 100 times lower than the protection threshold of 20 mV. The test enabled concluding that in line with the design criteria of the Cold Powering System, a fast discharge of any 2 kA corrector circuit does not trip the electrical protection of any other circuit in the system.

**Table 3:** Measured inductive coupling and self-inductance for the 18 kA, 15 kA and 2 kA corrector circuits.

Circuit	Test current (kA)	Ramp rate (A/s)	Nb_3_Sn Quadrupole	Nb-Ti Separation Dipole	Nb-Ti Trim	Nb-Ti Correctors
			Inductive coupling and self inductance (µH)			
Nb_3_Sn Quadrupole	18	100	31.0 Self-Inductance	8.1	15.0	2.3
Nb-Ti Separation Dipole	15	100	0.1	0.1 Self-Inductance	18.0	0.03
Nb-Ti Correctors	2	50	0.03	0.03	0.02	1.5 Self-Inductance

### Current leads

**Fig. 11:**
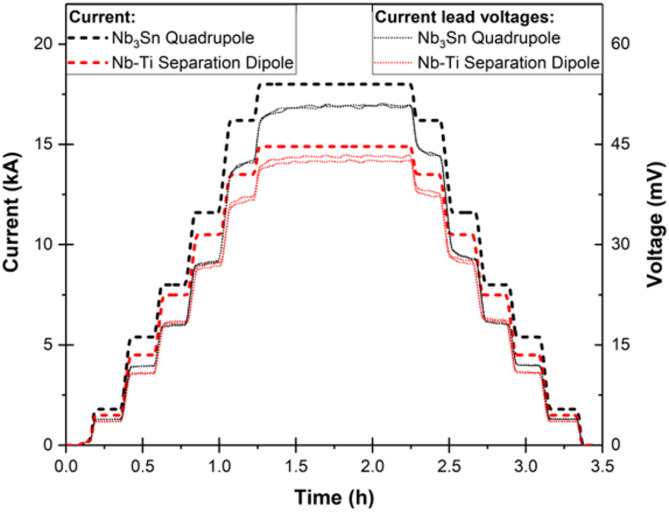
Voltage drop measured across the resistive parts of 18 kA and 15 kA current leads when operated at the test currents with T_HTS_ equal to 50 K and mass flow rates of 0.84 g/s and 0.69 g/s, respectively.

The current leads consist of a resistive part, which is a heat exchanger cooled by forced flow of helium gas, and of a REBCO HTS part. The design is similar to that of the LHC current leads^9^, with the difference that the HTS part consists of REBCO round cables^10^ instead of stacks of Bismuth-Strontium-Calcium-Copper-Oxide (BSCCO) 2223 silver-gold (Ag-Au) tapes. The helium gas enters the resistive part of the current leads at a temperature which is expected to be in between 25 K and 35 K: after having passed through the Superconducting Link and cooled the MgB_2_ to REBCO splices, which are at about 20 K, it cools the mechanical structure of the DFHX, the HTS parts of the current leads, the REBCO to copper splices at the cold end of the resistive heat exchanger, and it finally enters in and cools the resistive part of each current lead. The minimum mass flow rate for the resistive part of a current lead cooled with helium gas entering at about 25 K to 35 K is ranging from about 0.0048 g/s kA to 0.055 g/s kA^9^. The 18 kA and 15 kA current leads were able to operate at the test currents with the optimum rate. By design, the 18 kA and the 15 kA current leads are identical and optimized for operation at 18 kA. Figure 11 reports the voltage drop measured across the resistive parts of those current leads when operated at the test currents with T_HTS_ equal to 50 K and mass flow rates of 0.81 ± 0.03 g/s and 0.68 ± 0.01 g/s respectively. The helium flow rates are stable with time. The resistive heat exchangers of the 2 kA current leads are all identical, while the HTS part of the Trim circuit is designed for 7 kA in order to cope with electrical transients in the magnets and currents of up to 7 kA with no resistive transition. Measurements in steady state at 2 kA with T_HTS_ of 50–60 K resulted in mass flow rates of 111 ± 8 mg/s and 142 ± 8 mg/s, respectively. Differently from the 18 kA and 15 kA current leads, where each resistive part is surrounded by a vacuum insulation jacket, the 2 kA current leads are grouped in assemblies of four inside a common vacuum insulated envelope. Two of the four 2 kA current leads have an inclination with respect to ground of about 6 degrees, while the other two on the same flange are inclined by about 15 degrees. The difference in mass flow rate is attributed to thermal coupling in between the gas flowing inside the leads and the static gas stratified inside the common vacuum insulating envelope. The two current leads with a higher inclination are expected to be surrounded by stratified gas at a slightly (5 K to 10 K) higher temperature (density of helium gas increases as temperature decreases), which impacts on the global performance by demanding an increased flow rate. There are in total three assemblies of four 2 kA current leads: the behaviour of the leads in the different assemblies and with the same geometrical configuration is identical.

During operation no cold spots could be detected at any location along the currents leads, including the room temperature terminations where the room temperature cables are connected.

## Discussion

A 100 kA class DC superconducting system, based on MgB_2_ and REBCO technology, was designed, assembled and tested at CERN. The system successfully transferred a total DC current of up to |94| kA, the maximum current that could be delivered by the power converters, with MgB_2_ at up to 20 K and REBCO at up to 60 K, and it passed the electrical insulation tests at the target voltage of 2.3 kV under nominal operating conditions. This is the first of eight systems that will be installed in the LHC underground as from 2028 for operation at the start of the LHC High Luminosity Upgrade in 2030.

The Cold Powering System incorporates Nb-Ti cables in a saturated liquid helium bath at 4.5 K, a 74.5 m long Superconducting Link with MgB_2_ cables in the temperature range from 4.5 K up to about 20 K, REBCO cables from about 20 K up to 60 K, and current leads providing the electrical transfer to room temperature. It is the first transmission system that relies on long and high current (up to 18 kA individually) MgB_2_ cables. To our knowledge, the system transported the highest current ever reached in DC mode (|94| kA).

The choice of the MgB_2_ superconductor in the Superconducting Link was driven by the affordable cost of the conductor, its availability in kilometre lengths, its behaviour in case of resistive transition^11^ that enables use of conventional and reliable quench protection methods and electronics^7^, and the availability in the LHC of helium enabling operation at up to 20 K.

Because of the requirement of feeding several superconducting magnet circuits, the Cold Powering System incorporates a multiplicity of cables and current leads, nineteen in total, that are tightly arranged inside the Superconducting Link flexible cryostat and in the DFX and DFHX termination cryostats. This adds challenges to the design for maintaining compactness, dealing with the electrical insulation of each polarity, avoiding electro-magnetic cross talks among circuits, ensuring the mechanical flexibility required for installing and spooling the Superconducting Link, implementing the instrumentation necessary for protecting and operating each circuit individually, and fitting a multiplicity of electrical splices, among different superconducting cables, in compact volumes. A single polarity 120 kA or two polarities each rated at 60 kA, applicable to DC superconducting power transmission for instance, would fit with ease in a Superconducting Link cryostat with the same dimensions, but all listed complexities would not apply or would be significantly reduced.

From the point of view of cost and efficiency of the cryogenic cooling, the design of the system is such that the total helium mass flow rate corresponds to what is needed for operating the current leads. The Superconducting Link transfers both current and helium gas to the current leads, and the helium flow corresponds to that required for operating optimized current leads. To obtain this performance, a two-wall and low static heat load cryostat (≤ 2 W/m at any temperature in the range from 4.5 K to 20 K, with an external diameter of 220 mm and an inner diameter of 100 mm) was specifically developed for the Superconducting Link project in industry. The two-wall configuration was preferred to the four-wall configuration, which includes an actively cooled thermal screen, to enhance flexibility, simplify the design of the system and ease handling and installation aspects. The heat load of the Superconducting Link is mainly due to thermal radiation from room temperature to the cryogenic environment and thermal conduction through the vacuum insulation envelope, in between the two corrugated pipes, where MLI and spacers are located. Published values of static heat load for two-wall flexible cryostats with dimensions suitable for containing one, two or three electrical poles, are in the range of 1 W/m to 2.5 W/m at 77 K. These values are expected to increase by a factor of 4 to 6 with bends^12^. The 1.6 W/m at 4.5 K to 20 K of the Superconducting Link, operated in the convoluted geometry reported in Fig. 3, represents therefore a remarkable performance when compared to what before available in industry. It should be noted that the static heat load was measured with the MgB_2_ cables installed inside the cryostat. The design is such that the weight of the cables, which is of the order of 23 kg/m, did not impact on the thermal performance.

The current leads define the mass flow rate for the system. Optimized self-cooled current leads, operating between room temperature and liquid helium, conduct about 1.1 W/kA at 4.5 K, corresponding to a mass flow rate of about 0.055 g/s kA, i.e. 5.2 g/s at |94| kA, and a similar flow of about 0.055 g/s kA is required for cooling the resistive part of the current leads when helium gas enters at a temperature in the range from 25 K to 35 K. The full Cold Powering System is cooled by a flow of 0.052 g/s kA, i.e. 4.9 g/s at |94| kA: the change in enthalpy of the gas from 4.5 K to 20 K is used to cool the Superconducting Link, while the change of enthalpy of the gas from about 20 K to room temperature is used to cool the current leads. The system is optimized in such a way that the Superconducting Link does not add cryogenic cost to the cooling of the system.

**Fig. 12:**
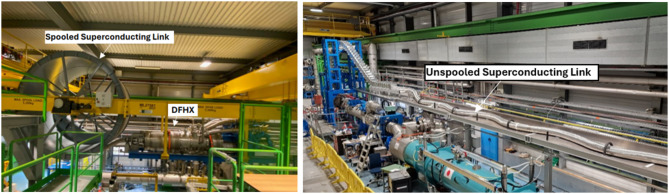
Transport of the Cold Powering System (left) and respooling after transport (right). The radius of the spool onto which the Superconducting Link is wound is 2 m.

By design the Superconducting Link can be wound onto a large spool (radius of 2 m) after having been connected to the DFHX with the current leads. This enables implementing the strategy adopted for the transport and for the installation of the system in the LHC underground: a Cold Powering System is assembled, tested in nominal operating conditions in the configuration reported in Fig. 3, spooled and finally transported. Such an operation has been successfully performed. Figure 12 left shows a Superconducting Link, attached to the DFHX, wound onto a spool and being transported after completion of the qualification tests. This is an example of a 100 kA class transmission line transported together with the electrical terminations, ready to be installed and connected to the room temperature cables. After re-spooling (Fig. 12 right), the system successfully passed the high voltage and leak tightness tests.

The developed system is cooled by a forced flow of helium gas generated inside the DFX. The cooling with gas is a reliable choice for a transmission system that has a long and convoluted geometry and that must operate, at a specific location, in a vertical configuration when it connects the HL-LHC new galleries to the LHC main tunnel. The use of MgB_2_ and REBCO ensures a large temperature margin, i.e. a margin between the nominal operating temperature and the maximum operating temperature just below the critical temperature of the superconductor at the design current, of at least 5 K for MgB_2_ and 10 K for REBCO. This margin is important since it eases cryogenic constraints and operation. Even if the nominal currents will never be exceeded during HL-LHC operation, by design the current margin for the MgB_2_ and the REBCO, i.e. the margin between the nominal current and the maximum transportable current just below the critical current of the superconductor at 20 K for MgB_2_ and at 60 K for REBCO, is at least 20%. Cooling of the high current MgB_2_ and REBCO splices with forced flow of helium gas was proven to be effective. The available temperature margin makes the operation of the splices robust and reliable. Use of helium gas is the natural choice for the HL-LHC, where helium is available for the magnets. The Superconducting Link could, however, operate at 20 K in liquid hydrogen. Work in this direction was done by^13^, where a 10 m long cable, in a cryostat with 40 mm external diameter, transported up to about 2.6 kA at 20 K. This cable was made with flat MgB_2_ tape, which was at the time the only geometry of ex-situ MgB_2_ conductor available in industry. The MgB_2_ round wire used in the Superconducting Link has been developed for the project in industry, in collaboration with CERN. To date, the total quantity of wire needed for the ten Cold Powering Systems, about 1500 km, has been produced. The HL-LHC Cold Powering Systems represent the first large scale application of MgB_2_ round wire, which is also for the first time successfully used in a large electrical transmission system.

The developed MgB_2_ technology finds applications for various uses in society. An initiative in this direction was taken by the IASS institute in Potsdam, under the scientific direction of Prof. Carlo Rubbia, where superconducting transmission was identified as an enabling technology for deployment of renewable electricity generation. The choice of MgB_2_ was associated with liquid hydrogen as coolant, with the goal of simultaneous transmission of electric power and hydrogen fuel. A demonstrator in this direction was done at CERN, in the context of a collaboration agreement between CERN and IASS, where a 20 kA MgB_2_ electrical transmission line was successfully constructed and qualified at 20 K^14^. This work continued with the BEST PATHS (acronym for “BEyond State-of-the-art Technologies for rePowering Ac corridors and multi-Terminal HVDC Systems”) project of the FP7 framework of the European Commission that demonstrated a DC monopole MgB_2_ cable system operated in helium gas at 10 kA/320 kV, corresponding to a transmitted power of 3.2 GW^15^.

The construction and qualification at CERN of the first Cold Powering System for HL-LHC demonstrate the feasibility and the potentials of very high DC current, 100 kA class, MgB_2_ based electrical transmission lines operated at up to 20 K.

## Conclusions

A complete system solution for a 100 kA class DC High Temperature Superconducting transfer line was developed and qualified at CERN. The system is cooled by a forced flow of helium gas. It incorporates MgB_2_ and REBCO superconductors operated at up to 20 K and 60 K, respectively. The system successfully transported up to |94| kA in DC mode, the maximum current that could be delivered by the power converters, and it successfully underwent steady state and transient tests representative of different operating modes in the LHC. While the system was developed for use in the LHC accelerator, it is a potential platform for wider energy applications including long power transmission lines and industrial applications that can benefit from a sustainable transfer of high currents, possibly at low voltage.

## Data Availability

The datasets used and/or analysed during the current study are available from the corresponding author on request.
